# Safety of the Transcranial Focal Electrical Stimulation via Tripolar Concentric Ring Electrodes for Hippocampal CA3 Subregion Neurons in Rats

**DOI:** 10.1155/2017/4302810

**Published:** 2017-08-13

**Authors:** Samuel Mucio-Ramírez, Oleksandr Makeyev

**Affiliations:** ^1^Laboratorio de Neuromorfología Funcional, Dirección de Investigaciones en Neurociencias, Instituto Nacional de Psiquiatría Ramón de la Fuente Muñiz, Calz. México Xochimilco No. 101, Col. San Lorenzo Huipulco, 14370 Mexico City, MEX, Mexico; ^2^Department of Mathematics, Diné College, 1 Circle Dr., Tsaile, AZ 86556, USA

## Abstract

Epilepsy is a neurological disorder that affects approximately one percent of the world population. Noninvasive electrical brain stimulation via tripolar concentric ring electrodes has been proposed as an alternative/complementary therapy for seizure control. Previous results suggest its efficacy attenuating acute seizures in penicillin, pilocarpine-induced status epilepticus, and pentylenetetrazole-induced rat seizure models and its safety for the rat scalp, cortical integrity, and memory formation. In this study, neuronal counting was used to assess possible tissue damage in rats (*n* = 36) due to the single dose or five doses (given every 24 hours) of stimulation on hippocampal CA3 subregion neurons 24 hours, one week, and one month after the last stimulation dose. Full factorial analysis of variance showed no statistically significant difference in the number of neurons between control and stimulation-treated animals (*p* = 0.71). Moreover, it showed no statistically significant differences due to the number of stimulation doses (*p* = 0.71) nor due to the delay after the last stimulation dose (*p* = 0.96). Obtained results suggest that stimulation at current parameters (50 mA, 200 *μ*s, 300 Hz, biphasic, charge-balanced pulses for 2 minutes) does not induce neuronal damage in the hippocampal CA3 subregion of the brain.

## 1. Introduction

Epilepsy is a neurological disorder that affects approximately one percent of the world population with up to three-fourths of all people with epilepsy living in developing countries [1]. Recently, electrical brain stimulation has shown promise to reduce seizure frequency but the best structures to stimulate and the most effective stimuli to use are still unknown [2].

Noninvasive tripolar concentric ring electrodes (TCREs) perform the second spatial derivative, the Laplacian, on the scalp potentials. Previously, it has been shown that tEEG, Laplacian electroencephalography (EEG) via TCRE configuration, is superior to conventional EEG with disc electrodes since the tEEG has significantly better spatial selectivity, signal-to-noise ratio, and mutual information [3]. Because of such unique capabilities, TCREs have found numerous applications in a wide range of areas including, in particular, seizure attenuation using transcranial focal stimulation (TFS) applied via TCREs [4–8]. Unlike electrical stimulation via conventional disc electrodes that is usually applied across the head, TFS via TCRE has a much more uniform current density and focuses the stimulation directly below the electrodes [9]. Previously, TFS has been shown to attenuate acute seizures [4] and reduce the convulsive expression and amino acid release in the hippocampus [5] during a pilocarpine-induced status epilepticus and reduce both electrographic [6, 7] and behavioral [7, 8] seizure activity in pentylenetetrazole-induced seizure model that is widely used for testing both seizure susceptibility and screening of new antiepileptic drugs [10].

Long-term goal for TFS is to control seizures, so safety has to be tested in animal models first. Previous work on safety testing of TFS includes assessment of the effect on scalp [11] and cortex tissue [12] as well as on memory formation [13] in rats. The effect of TFS via TCREs on rat scalp was quantitatively analyzed by calculating the temperature profile under the TCRE and the corresponding energy density with electrical-thermal coupled field analysis using a three-dimensional multilayer model [11]. Infrared thermography was used to measure skin temperature during TFS to verify the computer simulations. Besio et al. performed a histological analysis to study cell morphology and characterize any resulting tissue damage [11]. It was concluded that as long as the specified energy density applied through the TCRE was kept below 0.92 (A^2^/cm^4^ s^−1^), there was no significant damage to the rat scalp below the electrode. Another histomorphological analysis was performed to assess the effect of TFS via TCRE on rat cortical tissue (directly below the TCRE) [12]. Control, single-dose, and five-dose TFS-treated animals were evaluated at 24 hours, one week, and one month after the last administration of TFS. Integrated optical density (IOD) was measured with densitometry software. No statistically significant difference in IOD values was found for control and both groups of TFS-treated rat brains, and morphological analysis did not show any pyknotic neurons, cell loss, or gliosis that might confirm any neuronal damage to the cerebral cortex [12]. Finally, the effect of TFS on short- and long-term memory formation was assessed using object recognition test [13]. The results for naïve control and single-dose TFS-treated rats suggested that TFS has no adverse effect on the memory formation [13].

The goal of this study was to assess the possible effect of TFS on hippocampal CA3 subregion neurons in rats. The rational for the CA3 subregion analysis stems from it being one of the most important regions of the limbic system. In particular, studies suggest that the CA3 subregion of the dorsal hippocampus mediates the acquisition and encoding of spatial information within short-term memory with duration of seconds and minutes [14]. Moreover, CA3 mediates encoding of information requiring multiple trials to construct relational representations [14]. Neuroanatomical studies that have investigated the effects of different stimuli or manipulations suggest that even minimal damage to the neurons in this subregion may affect synthesis and production of different neurotransmitters such as glutamate and GABA [15, 16]. Electrical stimulation has been shown to cause dendritic sprouting [17], and our preliminary in vivo study [18] verified by the finite element method modeling [19] suggested that TFS may be sufficient to cause the activations of neurons in the hippocampus. Therefore, the objective of this study was to assess the significance of cell loss or, more generally, change in the number of neurons in the CA3 subregion due to TFS. While in our previous work IOD was measured [12], in this study, we used neuronal counting because it is an established approach to assess the degree of neuronal loss as a measure of healthy neuronal density in the homogeneous CA3 subregion [20] and therefore is a better fit to the objectives of this study.

## 2. Material and Methods

### 2.1. Animals

Male adult Sprague-Dawley rats (weighing 220–320 g) were used in this study (Harlan Laboratories Inc., Madison, WI). They were maintained under laboratory-controlled conditions (12 h/12 h normal light/dark cycle, 25°C) with food and water provided ad libitum. The care of all animals followed the standards set by the American Association of Laboratory Animal Care, and the experimental protocol was approved by the University of Rhode Island Institutional Animal Care and Use Committee.

### 2.2. Statistical Analysis

Statistical analysis was performed using Design-Expert software (Stat-Ease Inc., Minneapolis, MN, USA). Full factorial design of analysis of variance (ANOVA) was used with three categorical factors [21]. The first factor (A) was the presence of TFS stimulation presented at two levels corresponding to TFS-treated and control (sham TFS) animals. The second factor (B) was the time delay between the last TFS application and transcardial perfusion of the animal presented at three levels corresponding to 24 hours, one week, and one month. These delays were incorporated to observe the time course of any possible injuries to the CA3 subregion. The third factor (C) was the number of TFS applications presented at two levels corresponding to a single dose of TFS and five doses of TFS administered every 24 hours for five consecutive days. Factor C allows observing the effect of TFS when applied acutely or repeatedly. The response variable was the neuronal counting data (described below) measured in a separate group of animals (*n* = 3) for each of the 2 × 2 × 3 = 12 combinations of levels of three factors (grand total of *n* = 36 for all 12 groups). The full factorial design of our study is presented in Table 1.

### 2.3. Application of TFS

On the day of the experiment, the rat's scalp was shaved and prepared with NuPrep abrasive gel (D. O. Weaver, Aurora, CO). The rat was held by one researcher while another placed a TCRE with conductive paste (1 mm Ten20, Grass Technologies, West Warwick, RI) on the scalp centered on the top of the head behind the eyes and in front of the ears. The TFS (50 mA, 200 *μ*s, 300 Hz, biphasic, charge-balanced pulses) was then applied for 2 minutes between the outer ring and central disc of the TCRE (with the middle ring floating). All the control animals were fully instrumented like the treated animals but received a single or five doses of sham TFS (0 mA).

### 2.4. Perfusion Protocol and Imaging

On the day of the perfusion, all rats were weighed and deeply anesthetized with a mixture of ketamine (80 mg/kg) and xylazine (12 mg/kg) i.p. They were transcardially perfused with 150 ml of heparinized saline solution (9%) followed by a 4% paraformaldehyde (Sigma Chemical Co., St. Louis, MO) solution in phosphate-buffered saline (PBS) 0.1 M, pH 7.4 through a perfusion pump at a flow rate of 900 ml/hour. After 30 minutes of perfusion, the brains were removed from the skull and immersed in 4% paraformaldehyde overnight. Then the fixative was discarded, and the brains were immersed in a sucrose (Sigma Chemical Co., St. Louis, MO) solution of 30% (in PBS 0.1 M, pH 7.4). The brains were kept refrigerated at 4°C until cut. Coronal sectioning was performed at 30 *μ*m (UltraPro 5000 cryostat vibratome). Every fifth section (1-in-5 series) containing the dorsal hippocampal CA3 subregion was collected (in PBS 0.1 M). The slices were stored at 4°C for later use. Tissue sections containing the region of interest were mounted on gelatinized slides and allowed to dry. Control and TFS-treated slides were Nissl stained the same day at the same time. The slices were dehydrated with serial alcohols (70%, 80%, 96%, and 100%), cleared with Xilens (Fisher Scientific, Hampton, NH), and mounted with Permount mounting media (Fisher Scientific, Hampton, NH).

### 2.5. Neuronal Counting

**Figure 1:**
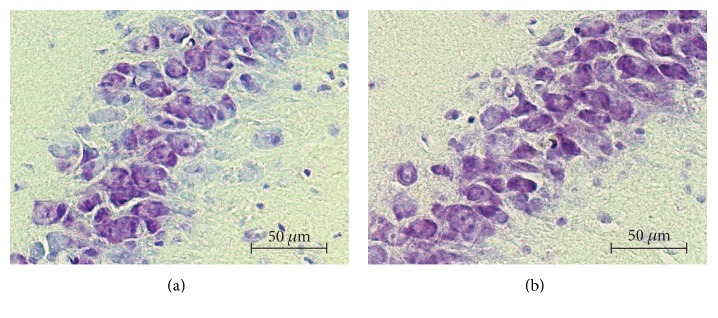
Examples of single fields of the coronal brain slices from the dorsal hippocampus CA3 subregion of: (a) control rat perfused 1 month after a sham TFS; (b) TFS-treated rat perfused 1 month after a single dose of TFS.

Brain slices for three sections of each brain in the CA3 subregion of the dorsal hippocampus were photographed with a microscope (Carl Zeiss, Oberkochen, Germany) equipped with a digital camera (Digital Sight DS-U3/DS-Fi1, Nikon Co., Japan) at 40x magnification. For consistency, we photographed only brain slices in the bregma interval −3.3 mm to −3.8 mm since this was the nearest region to where the TCRE was placed [22]. Then, we selected 3 adjacent fields containing the CA3 subregion for each brain slice. We repeated this for 4 slices resulting in 12 images for each brain. Neuronal counting was performed using ImageJ software (National Institutes of Health; http://imagej.nih.gov/ij). The counting field containing CA3 neurons was a rectangle of 220 *μ*m width and 165.38 *μ*m length. Examples of counting fields for representative control and TFS-treated animals perfused 1 month after a single dose of TFS (groups 5 and 6 from Table 1, resp.) are presented in Figure 1(). Since cell counting was performed manually, in order to avoid counting the same neuron more than once, we used a grid (140 rectangular sections of equal size). For cell counting, we defined neurons as circular cell bodies with an evident nucleus revealed by the Nissl technique. Final neuronal counting data was expressed in the number of neurons per *μ*m^2^ (neurons/*μ*m^2^). The adjacent fields for each slice were added together resulting in four neuronal counting values for each brain or 12 values for each of the 12 groups used in the statistical analysis.

## 3. Results

**Figure 2:**
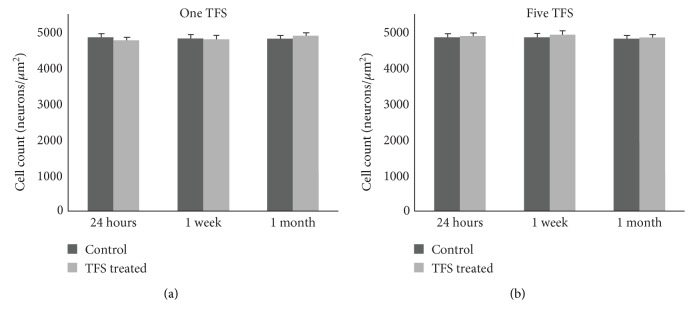
Numbers of neurons per *μ*m^2^ (mean and standard error) in the CA3 subregion in control and TFS-treated subgroups of the single-dose TFS (a) and five-dose TFS (b) groups.

Neuronal counting results obtained in this study for 12 groups are presented in Table 1 as numbers of neurons per *μ*m^2^ (mean ± standard error) and illustrated in Figure 2.

The ANOVA did not show any statistically significant effects in the model neither for the main factors A, B, and C nor for their interactions: A (d.f. = 1, *F* = 0.14, *p* = 0.71), B (d.f. = 2, *F* = 0.05, *p* = 0.96), C (d.f. = 1, *F* = 0.14, *p* = 0.71), AB (d.f. = 2, *F* = 0.5, *p* = 0.61), AC (d.f. = 1, *F* = 0.37, *p* = 0.54), BC (d.f. = 2, *F* = 0.2, *p* = 0.82), and ABC (d.f. = 2, *F* = 0.42, *p* = 0.66).

## 4. Discussion

The main result of this study is that TFS via TCREs at current stimulation parameters (50 mA, 200 *μ*s, 300 Hz, biphasic, charge-balanced pulses for 2 minutes) does not induce neuronal damage in the hippocampal CA3 subregion of the brain when applied acutely or repeatedly. The neuronal cell counting showed no statistically significant effect on the number of neurons due to such factors as the presence of TFS (factor A), the time delay after the last TFS application (factor B), and the number of TFS applications (factor C) as well as to all the factor interactions. Observed healthy neurons stained with cresyl violet were robust in shape and had a pale and spherical or slightly oval nucleus and a single large nucleolus (Figure 1()). The cytoplasm of the neurons could also be seen clearly, characteristics observed in all the animals (*n* = 36; Figure 1()).

These results are in line with our previously obtained results assessing the effect of TFS via TCRE on the scalp, cortex, and memory formation, further suggesting that TFS is safe as well as effective, at least in rats [11–13, 23]. Unlike the original study on memory formation [13] that assessed only the effect of a single dose of TFS, in this study, we also assessed the effect of five doses of TFS administered every 24 hours. Five daily doses were selected for chronic TFS application in this study as well as in our previous studies on cortical integrity [12] and memory formation [23] for consistency purposes because the same number of doses is commonly used in studies on both antiepileptic [24] and proepileptic [25] effects of drugs. Since long-term goal is to use TFS in clinical practice, the application of TFS via TCREs may need to be given more than once. Previously, we demonstrated the feasibility of an automatic noninvasive seizure control system in rats with pentylenetetrazole-induced seizures. A single dose or two doses of TFS were administered via TCRE where TFS was triggered automatically by a real-time tEEG-based electrographic seizure activity detector [26]. Therefore, the safety of TFS had to be evaluated for repeated TFS applications.

One of the limitations of the current study is that we did not have the resources to randomize the run order of the full factorial study design. Randomization could have helped balancing out the effect of nuisance factors [21]. Instead, processing of all the groups in Table 1 has been started simultaneously. Other assumptions of ANOVA including normality, homogeneity of variance, and independence of observations were verified ensuring the validity of the analysis with only one outlier studentized residual (0.7% of the total number) falling outside the [−3, 3] range [21].

Another limitation is that this study only assessed the effect of a single set of predefined TFS parameters (50 mA, 200 *μ*s, 300 Hz, biphasic, charge-balanced pulses for 2 minutes) rather than examining a large range of the stimulation parameters. In particular, adding a TFS-treated group with a set of parameters that causes histopathological effects could have helped establish the safety limits for TFS as well as validate the sensitivity of the safety testing approach used in detection of negative effects of stimulation. The reasoning behind using a single set of TFS parameters was threefold. First, the same set of parameters has been proven effective in attenuating acute pilocarpine- and pentylenetetrazole-induced seizures [4, 6–8, 26] and our aim was to keep the safety testing studies [12, 13, 23] consistent with the studies assessing the anticonvulsant effect of TFS. For example, in a previous study [4], a range of TFS frequencies (200, 300, 500, and 750 Hz), two pulse durations (200 and 300 *μ*s), and two current intensities (50 and 60 mA) were tested in an assessment of the effect of TFS on pilocarpine-induced status epilepticus in rats using a ramp stimulation protocol and no combination of TFS parameters yielded significantly better results than the parameter set used in the current study. Second, adding more factors such as TFS frequency, pulse duration, and current intensity would have further expanded our full factorial design resulting in an increase in the number of animals from currently used *n* = 36. Taking into account limited resources available for this study and the fact that recent results suggest anticonvulsant effect of TFS at currents much lower than the one used in this study (5 mA versus 50 mA) [27], the current set of ANOVA factors was selected as the most relevant one to the scope of the study. Finally, validity of the neuronal counting as an approach to assess the degree of neuronal loss in CA3 subregion stems from the fact that it is established and widely used both for hippocampus areas in general [28, 29] and CA3 in particular [20]. In a similar way, an established approach such as object recognition test was used in [13] without validating its sensitivity using a group of animals whose short- and long-term memory formation has been modified by TFS.

Since no significant changes due to TFS were observed using neuronal counting at this point of time, we did not perform further tests such as quantizing neuronal death and changes in neurotransmitters.

While it is difficult to draw a direct comparison between safety testing studies performed for other invasive or noninvasive brain stimulation techniques and the current study, an overview of the work of others in humans or animal models is presented below. For invasive deep brain stimulation (DBS), no significant tissue damage has been found in the brains of eight patients with Parkinson's disease treated with DBS continuously for up to 70 months [30]. All brain samples showed well-preserved neural parenchyma and only mild gliosis due to reactive changes to the surgical placement of the electrode. Although TFS is noninvasive, in our previous work, morphological analysis did not show any pyknotic neurons, cell loss, or gliosis in TFS-treated rat brains and there was no significant damage found in the current study [12].

Electroconvulsive therapy (ECT), a form of noninvasive brain stimulation, produces an adverse neurocognitive secondary effect in the form of memory dysfunction due to the disruption of specific brain regions [31]. Previously, it has been demonstrated that TFS does not produce adverse effects in the short- and long-term memory formation in rats [13, 23]. At the same time, in animal studies that used an electroconvulsive shock stimulus intensity and frequency comparable to human ECT, no neuronal loss has been shown by quantitative cell counts even after prolonged courses of treatment [32]. Similar approach was used to assess the safety of TFS in this study.

For other noninvasive brain stimulation techniques such as transcranial direct current stimulation (tDCS) or transcranial magnetic stimulation (TMS) and repetitive TMS (rTMS), guidelines with suggested stimulation parameter ranges for safe application exist based, in particular, on results of histomorphological studies in animal models [33–35]. Similar quantification of the range of TFS parameters allowing safe application of TFS via TCREs is among the objectives of our future work. Other objectives include determining the specific mechanisms of action of TFS via TCREs and investigating how the results in rats may translate to human epilepsy.

## 5. Conclusions

In this study, microscopic image analysis was used to conduct a safety test for a brain stimulation protocol assessing the possible effect of transcranial focal stimulation via tripolar concentric ring electrodes on hippocampal CA3 subregion neurons in rats. Neuronal counting was used to assess the significance of the effects of the presence of stimulation (treated animals versus controls), the number of stimulation doses (one versus five), and the delay after the last stimulation dose (24 hours, one week, and one month) on the number of neurons. Analysis of variance showed no statistically significant effects in the model suggesting the safety of the stimulation at current stimulation parameters (50 mA, 200 *μ*s, 300 Hz, biphasic, charge-balanced pulses for 2 minutes) for hippocampal CA3 subregion neurons.

## Figures and Tables

**Table 1:** Full factorial design of analysis of variance with the neuronal counting results.

Group	Categorical factors			Number of neurons per *μ*m^2^ (mean ± standard error)
	A: presence of TFS	B: time delay after the last TFS application	C: number of TFS applications	
1	TFS treated	24 hours	1	4785 ± 90.8
2	Control	24 hours	1	4862.5 ± 100.6
3	TFS treated	1 week	1	4805 ± 91.8
4	Control	1 week	1	4847.5 ± 84.2
5	TFS treated	1 month	1	4925 ± 74.3
6	Control	1 month	1	4842.5 ± 74.4
7	TFS treated	24 hours	5	4835 ± 96
8	Control	24 hours	5	4812.5 ± 67.8
9	TFS treated	1 week	5	4917.5 ± 138.9
10	Control	1 week	5	4800 ± 119.3
11	TFS treated	1 month	5	4800 ± 49.5
12	Control	1 month	5	4785 ± 68.3
